# Electrochemical Deposition of Ferromagnetic Ni Nanoparticles in InP Nanotemplates Fabricated by Anodic Etching Using Environmentally Friendly Electrolyte

**DOI:** 10.3390/nano12213787

**Published:** 2022-10-27

**Authors:** Călin Constantin Moise, Geanina Valentina Mihai, Liana Anicăi, Eduard V. Monaico, Veaceslav V. Ursaki, Marius Enăchescu, Ion M. Tiginyanu

**Affiliations:** 1 Center for Surface Science and Nanotechnology, University Politehnica of Bucharest, 313 Splaiul Independentei, 060042 Bucharest, Romania; 2 S.C. NanoPRO START MC S.R.L., Mitropolit Antim Ivireanu Street 40, 110310 Pitesti, Romania; 3 National Center for Materials Study and Testing, Technical University of Moldova, Bd. Stefan cel Mare 168, 2004 Chisinau, Moldova; 4 Academy of Sciences of Moldova, 2001 Chisinau, Moldova; 5 Academy of Romanian Scientists, 54 Splaiul Independentei, 050094 Bucharest, Romania

**Keywords:** anodization, neutral electrolyte, porous, electrochemical deposition, Ni nanoparticles

## Abstract

Porous InP templates possessing a thickness of up to 100 µm and uniformly distributed porosity were prepared by anodic etching of InP substrates exhibiting different electrical conductivities, involving an environmentally friendly electrolyte. Ni nanoparticles were successfully directly deposited by pulsed electroplating into prefabricated InP templates without any additional deposition of intermediary layers. The parameters of electrodeposition, including the pulse amplitude, pulse width and interval between pulses, were optimized to reach a uniform metal deposition covering the inner surface of the nanopores. The electrochemical dissolution of *n*-InP single crystals was investigated by measuring the current–voltage dependences, while the Ni-decorated *n*-InP templates have been characterized by scanning electron microscopy (SEM) and energy-dispersive X-ray spectroscopy (EDX). The proposed technology is expected to be of interest for sensing and photocatalytic applications, as well as for the exploration of their plasmonic and magnetic properties.

## 1. Introduction

Various porous templates, especially those with ordered arrangement of pores, have found a series of applications in nanofabrication [1]. Two types of templates are most widely used for nanofabrication purposes nowadays, namely porous alumina templates produced by the anodization of aluminum foils [2,3], and etched ion track membranes based on either inorganic materials or organic polymers [4,5]. In recent years, semiconductor nanotemplates have emerged as a prospective basis for the templated fabrication of nanowires and nanotubes from various materials, as well as of composite nanomaterials [6]. Wide possibilities to control the electrical conductivity of semiconductor templates, e.g., by means of external illumination and applied electric fields, are among their most important advantages, especially when the preparation of metallic nanotubes with a controlled diameter and thickness of the walls is envisaged.

Usually, the pores in semiconductors are introduced via anodization in electrolytes containing acids such as HF, HCl, H_2_SO_4_, HNO_3_, etc., or in alkaline electrolytes [7,8]. In the last decade, to make the process of nanofabrication based on anodic etching broadly accessible and environmentally friendly, research has focused on nanostructuring in neutral electrolytes based on an aqueous solution of NaCl, instead of the commonly used aggressive acids or alkaline electrolytes, for the purpose of the electrochemical nanostructuring of semiconductor substrates. Anodization in neutral electrolytes has proven to be feasible for the nanostructuring of III-V and II-VI semiconductor compounds, e.g., InP [9,10,11], GaAs [12,13], CdSe [12], and GaN [14,15]. Moreover, according to photoluminescence investigations, the obtained porous layers demonstrated no decrease in the near-bandgap emission intensity. This suggests that the surface recombination rate does not increase in porous templates, which is indicative of an effective passivation of the huge internal surface of the porous samples during anodization in the NaCl-based electrolyte.

Metallic nanotubes are widely used in optoelectronics and magnetic material applications. The optoelectronic applications are essentially based on surface plasmon polaritons excited and propagated on the extended dielectric/metal interface, which allow the manipulation and transmission of light at the nanoscale [16,17]. As concerns the magnetic materials, ferromagnetic nanowires, nanotubes and core–shell tubular arrays present considerable interest in high-density data storage, microelectronics and spintronics [18,19,20,21,22]. It was recently proposed to use GaAs nanowires, prepared by a cost-effective electrochemical etching procedure applied to GaAs substrates, as cores for the fabrication of ferromagnetic core–shell tubular arrays [23,24,25]. It was shown that such structures present interest from the point of view of possibilities to choose the direction of the axis of ferromagnetic nanotubes constituting the shell oriented either in-plane or out-of-plane with the GaAs substrate. The interest in such structures is related to their pronounced magnetic anisotropy. 

It was found that the magnetic anisotropy of nanowire and nanotube arrays is strongly influenced by their geometrical parameters, such as the diameter, length, and nanotube wall thickness [26,27,28]. As concerns ferromagnetic nanotubes deposited on GaAs nanowires, their diameter demonstrated to date is larger than 200 nm. InP is another III-V material suitable for the preparation of porous templates by means of anodization. Successful deposition of Au nanotubes inside the pores of InP templates has been previously reported [6,9,11]. 

The goal of this paper is to demonstrate the fabrication of InP templates with the diameter of pores controlled by the conductivity of as-grown InP substrates involving the anodization process, allowing the formation of nanotubular structures and the further direct electrochemical deposition of ferromagnetic Ni nanoparticles (NPs) inside the pores without any preliminary functionalization of the pore walls.

## 2. Materials and Methods

Crystalline 500-μm thick *n*-InP(100)-oriented substrates with the free electron concentration of 2 × 10^17^ cm^−^^3^ and 2 × 10^18^ cm^−^^3^, supplied by University Wafer, Boston, MA, USA, were used in this study. Anodization of *n*-InP crystals was carried out in 1.75 M and 3.5 M NaCl aqueous solutions for 180 s, in a potentiostatic mode, under different applied voltages, their selection being based on the I-V curves. The anodization was performed in a common two-electrode cell, where the sample served as a working electrode as described elsewhere [29]. Briefly, an electrical contact with a conductive silver paste was made on the backside of the samples. From the top side, the sample was pressed by an O-ring to leave a surface with an area of 0.2 cm^2^ exposed to the electrolyte. A mesh of platinum wire with 0.5 mm diameter was used as the counter electrode. An AUTOLAB Potentiostat/Galvanostat/EIS/ECD (Metrohm Autolab B.V., Utrecht, The Netherlands) was used to record the polarization curves with a scan rate of 10 mV·s^−1^ before the electrochemical etching, as well as chronoamperometry curves recorded during the anodization process. After the pore growth, the top nucleation layer of porous InP samples was removed by isotropic wet etching, immersing the specimen in 1:1 (vol.) HCl:H_3_PO_4_ mixture for 25 s, followed by sonication in acetone for 60 s.

The Ni NPs electroplating in *n*-InP template was performed by pulsed electrochemical deposition involving the same two-electrode electrochemical cell, where the sample served as the working electrode and the Pt mesh acted as the counter electrode. A source meter, Keithley 2400 (Tektronix, Beaverton, OR, USA) was used for rectangular pulse generation with a voltage amplitude of −20 V. The electrodeposition process was carried out at 45 °C, employing an aqueous electrolyte containing Ni(SO_3_NH_2_)_2_·4H_2_O (400 g·L^−1^), NiCl_2_·6H_2_O (12 g·L^−1^), H_3_BO_3_ (40 g·L^−1^), and CH_3_(CH_2_)_11_OSO_3_Na (0.5 g·L^−1^), with a pH value of 3.5.

The morphology of the prepared samples was studied using a Hitachi SU8230 Scanning Electron Microscope (SEM) (Hitachi High-Tech Corp., Tokyo, Japan) equipped with an Energy Dispersive X-Ray Spectroscopy (EDX) detector-analyser (Oxford Instruments PLC, Oxford, UK).

## 3. Experimental Results and Discussion

### 3.1. Optimization of the Anodization Process

**Figure 1:**
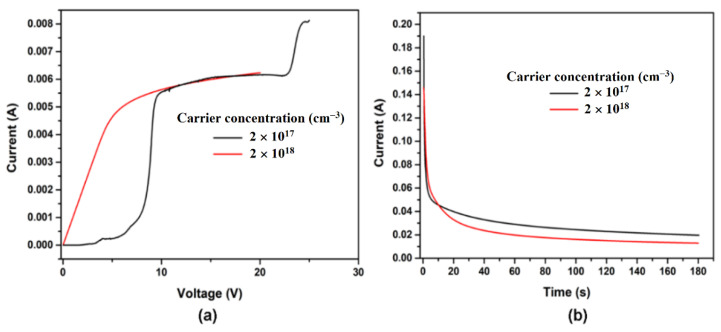
(**a**) The polarization I-V curves measured with a scan rate of 10 mV·s^−1^ before the anodization of InP samples with a free carrier concentration of 2 × 10^18^ cm^−3^ (red) and 2 × 10^17^ cm^−3^ (black). (**b**) Chronoamperograms recorded during InP templates fabrication with a carrier concentration of 2 × 10^18^ cm^−3^ (red) and 2 × 10^17^ cm^−3^ (black) at 6.5 V and 20 V, respectively.

The electrochemical dissolution behavior of *n*-InP crystals with different electron concentrations in 3.5 M NaCl electrolyte was characterized by I-V curves, as shown in Figure 1a. The current sharply increased up to an applied potential of 6 V for the sample possessing higher conductivity, with a concentration of 2 × 10^18^ cm^−3^ (red curve in Figure 1a). During the further increase of the applied voltage, a current plateau was noticed. The use of crystals with the free electron concentration of 2 × 10^17^ cm^−3^ determined a different evolution of I-V curves (black curve in Figure 1a). Therefore, a small increase of the current up to 8 V was evidenced, with the etch pit formation at about 5 V. The voltage domain between 12–22 V presents interest for a controllable pore growth. At higher applied voltages (more than 22 V), fluctuations in pore diameters or even electropolishing are expected. The recorded behavior of the current against time shows an exponential decrease, as can be observed in Figure 1b, and can be related to the depletion of the electrolyte in pores and difficulties of electrolyte exchange with the deepness of the produced porous layer.

**Figure 2:**
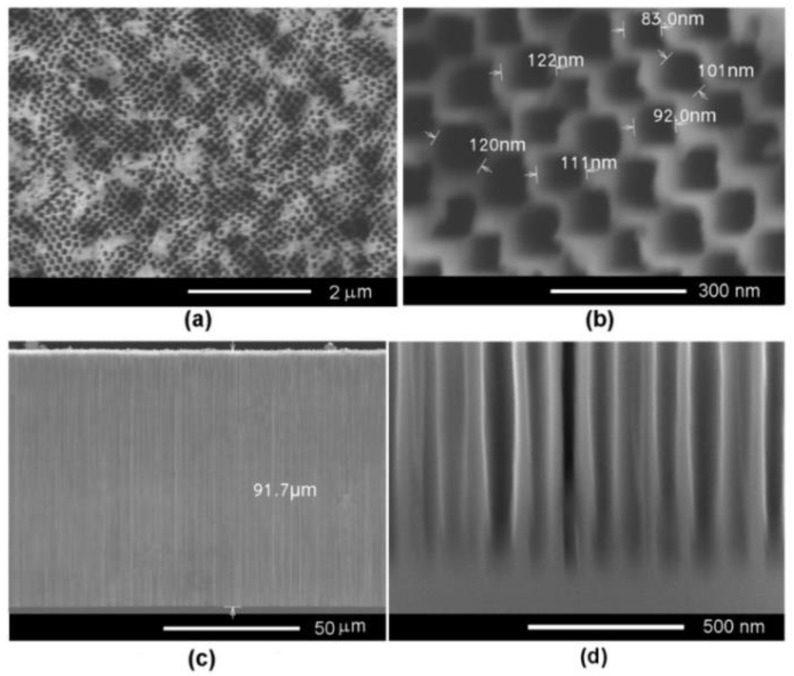
(**a**) SEM front view of a porous template prepared on InP with a carrier concentration of 2 × 10^18^ cm^−3^ by anodic etching in 3.5 M NaCl solution at an applied voltage of 6.5 V. (**b**) Zoomed-in view. (**c**) Cross-section view. (**d**) Zoomed-in view of the region at the bottom of the pores presented in (**c**).

As mentioned above, the higher the carrier concentration in the semiconductor material, the lower the anodization voltage necessary for achieving a uniform and controlled porosification. As shown in Figure 2, an anodization voltage of 6.5 V is enough to produce templates presenting a uniform distribution of pores in InP substrates with a carrier concentration of 2 × 10^18^ cm^−3^. 

Pore diameters between 80–120 nm have been evidenced when applying this anodization voltage associated with the self-ordered arrangement of pores. Note that self-ordering of pores occurs due to the growth of current line-oriented pores and without any photolithographic means [6]. The thickness of the porous layer is around 100 µm, and the pores maintain their diameter along the pore propagation direction, which is perpendicular to the sample surface.

**Figure 3:**
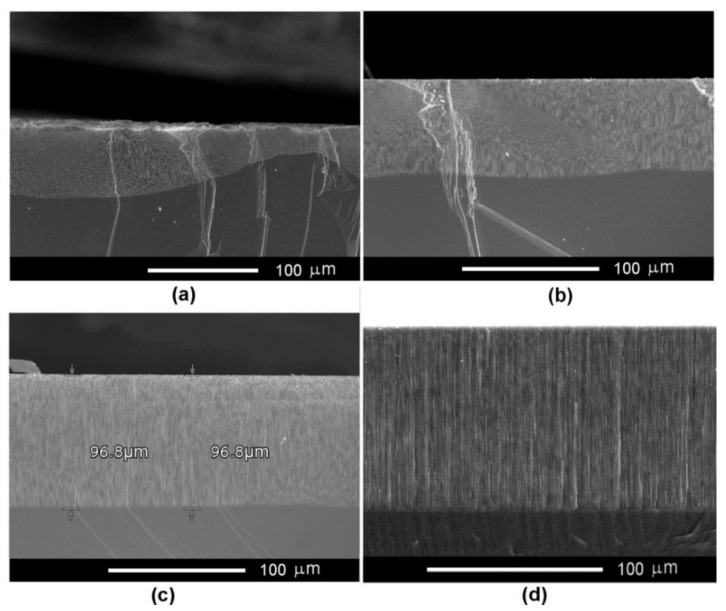
SEM cross-sectional view of porous template prepared on InP with a carrier concentration of 2 × 10^17^ cm^−3^ by anodic etching in 3.5 M NaCl electrolyte at applied anodization voltage of (**a**) 10 V, (**b**) 12 V, (**c**) 15 V, and (**d**) 20 V.

The InP substrates possessing a carrier concentration of 2 × 10^17^ cm^−3^ require anodization voltages higher than 10 V, as can be seen from the I-V curve in Figure 1a. Figure 3 illustrates the optimization of the anodization voltage in these substrates when they are electrochemically etched in 3.5 M NaCl electrolyte. The thickness of the produced porous layer exhibits a strong fluctuation at the applied voltage of 10 V (see Figure 3). The fluctuations are significantly reduced at 12 V (Figure 3b), and the thickness of the porous layers is constant at 15 V and 20 V (Figure 3c,d). The nonuniformity in the thickness of the porous layer at low applied voltages can be explained by the preponderant growth of the crystallographic oriented pores characterized by radial propagation in directions underneath the surface.

**Figure 4:**
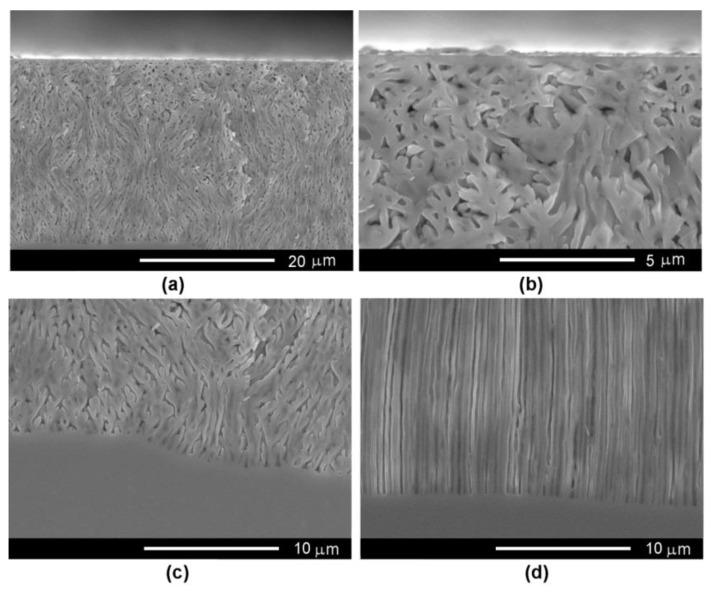
(**a**) SEM cross-sectional view of a porous template prepared on InP with a carrier concentration of 2 × 10^17^ cm^−3^ by anodic etching in 1.75 M NaCl electrolyte at applied voltage of 12 V; (**b**) The image of the region at the surface of the sample; (**c**,**d**) SEM cross section view at the bottom of pores for a porous template prepared on InP with a carrier concentration of 2 × 10^17^ cm^−3^ by anodic etching in (**c**) 1.75 M NaCl and (**d**) 3.5 M NaCl electrolyte at applied voltage of 12 V.

The concentration of the electrolyte solution also influences the produced porosity, as presented in Figure 4. One can see that a flower-like morphology of the pores is developed using the 1.75 M NaCl electrolyte, at an applied anodization voltage of 12 V (Figure 4a–c). This behavior is characteristic for anodization in low-concentration electrolytes, resulting in nucleation of the pores at surface defects (dislocations) with subsequent radial growth facing away underneath the surface. On the other hand, the pores propagate perpendicularly to the sample surface when anodization is performed at the same voltage in the 3.5 M NaCl electrolyte, as illustrated by the SEM image taken at the bottom of the pores (Figure 4d). In such a case, the dissolution mechanism occurs as follows: the formation of pore nuclei at surface with a further abundant branching of crystallographically oriented (crysto) pores forming the nucleation layer (see Section 3.2). Around each pore, a surface-depleted layer (W) is formed with a thickness that can be estimated from the relation (1):

(1) $$W=\sqrt{\frac{2\cdot \varphi_{0}\cdot \varepsilon_{0}\cdot \varepsilon_{s}}{q\cdot N_{D}^{+}}}$$

where *φ*_0_ is the surface potential, *ε_0_ε_S_* is the static dielectric constant of the material, and *N_D_^+^* is the concentration of ionized donors.

At a certain stage of pore growth in the depths, the branching of crysto pores slows down and the transition from crysto pores to current line-oriented (curro) pores occurs. The current line-oriented pores start to grow aligned perpendicular to the substrate surface. It should be mentioned that current line-oriented pores cannot intersect with each other, therefore providing conditions for the self-ordering of pores [6,30]. Taking into account that around each pore a depletion layer is formed, and the fact that curro pores cannot intersect, they start to push each other to maintain the two depletion layers 2 W between them [6].

**Figure 5:**
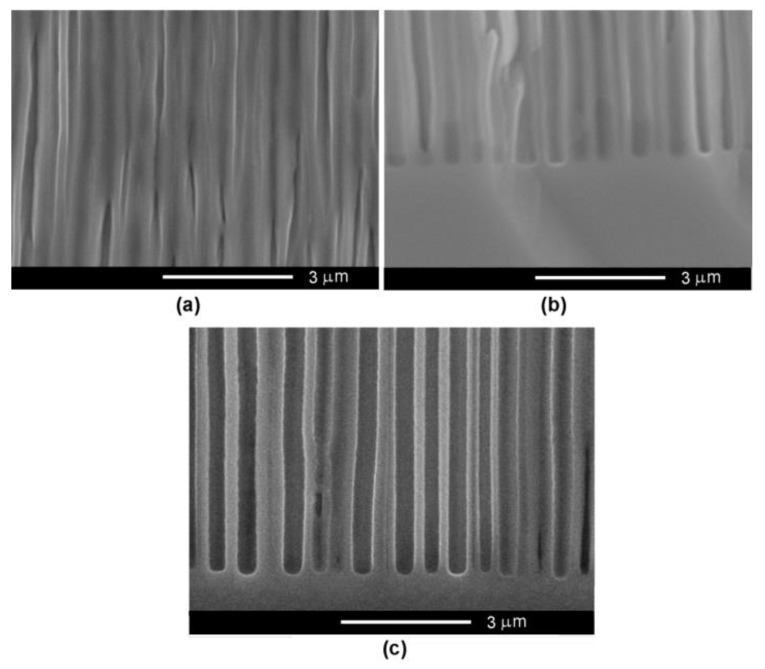
SEM cross-sectional view of a porous template prepared on InP with a carrier concentration of 2 × 10^17^ cm^−3^ by anodic etching in 3.5 M NaCl solution at different applied voltages: (**a**) 12 V, (**b**) 15 V, and (**c**) 20 V.

The uniformity of the pore diameters along the propagation direction improved as the anodization voltage increased from 12 V to 20 V, as shown in Figure 5. Thus, one can conclude that the use of the 3.5 M NaCl electrolyte is suitable for the anodization of InP substrates possessing a carrier concentration of 2 × 10^17^ cm^−3^, while the optimum anodization voltage is 20 V. However, removal of the top nucleation layer is necessary for opening the entrance in pores, which is necessary for templated metal deposition.

### 3.2. Optimization of the Pore Opening Process

**Figure 6:**
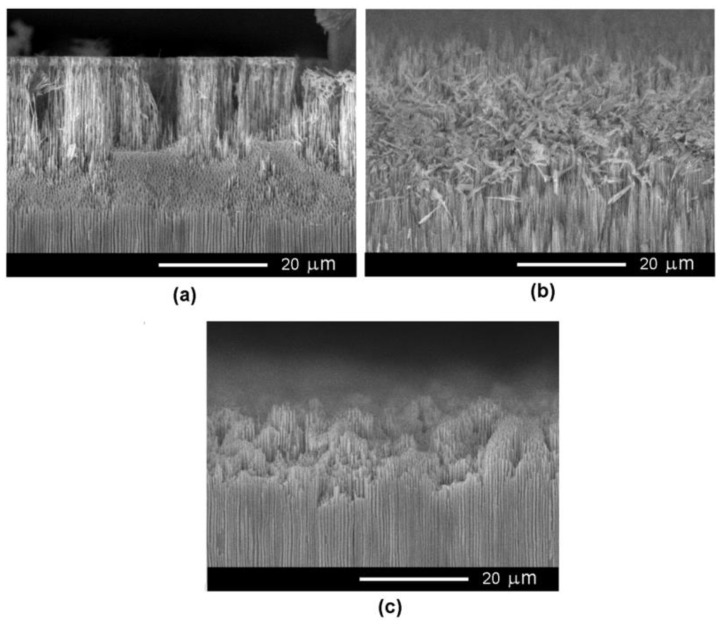
(**a**) SEM cross-sectional view of a porous template prepared on InP with a carrier concentration of 2 × 10^17^ cm^−3^ by anodic etching in a 3.5 M NaCl electrolyte at an applied voltage of 20 V before pore opening; (**b**) the same sample after chemical treatment in HCl:H_3_PO_4_ (1:1) for 25 s; (**c**) the same sample with pore opening by sonication during 1 min in acetone.

In both samples characterized by different free carrier concentrations, the top nucleation layer formed with a thickness of about 2–5 µm. It should be noted that in the sample with a lower electrical conductivity, the ramification of crystallographic oriented pores occurred slower, and the self-ordering process, characterized by strictly parallel pores with an equal wall thickness, occurred at a deepness of about 15 µm. Consequently, a perfect cross-sectioning of the porous layer is difficult to obtain (see Figure 6a). Generally, for samples with 2 × 10^18^ cm^−3^ carrier concentration, it is enough to use chemical dissolution for 20–25 s. At the same time, the nucleation layer in the sample with a carrier concentration of 2 × 10^17^ cm^−3^ requires a longer duration of wet isotropic chemical etching allowing the dissolution of thick pore walls, in comparison with the high carrier concentration sample. It is obvious that during a longer duration of chemical etching, the electrolyte will act more deeply. Consequently, to avoid the complete removal of the porous layer, a combination of chemical etching for 25 s in an acidic solution followed by sonication in acetone was proposed for removal of the top nucleation layer and opening the entrance of the pores. In such a way, the pore walls are not dissolved but are destroyed by ultrasound. One can see from Figure 6b that the top porous layer is destroyed when the sample is chemically treated in an acid solution for 25 s. The final removal of the nucleation layer and the opening of pores is obtained by sonication during 1 min in acetone (Figure 6c).

**Figure 7:**
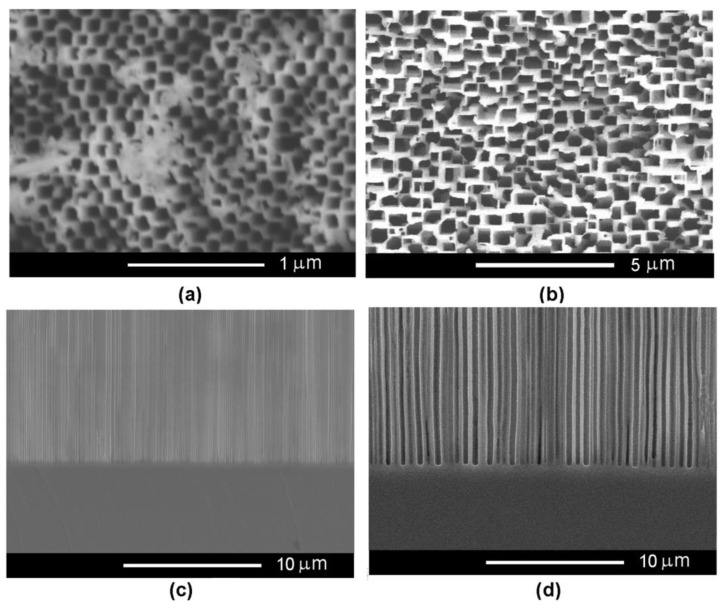
SEM front view of a porous template prepared on InP with a carrier concentration of 2 × 10^18^ cm^−3^ (**a**) and 2 × 10^17^ cm^−3^ (**b**) after the pore-opening process; (**c**,**d**) SEM cross-sectional views of porous template prepared on InP possessing a carrier concentration of (**c**) 2 × 10^18^ cm^−3^ and (**d**) 2 × 10^17^ cm^−3^.

Figure 7 compares two porous templates with opened pores prepared on InP possessing different carrier concentrations. One can see that the dimensions of the pores increased about 5 times from around 100 nm to around 500 nm as the carrier concentration in the InP substrate decreased from 2 × 10^18^ cm^−3^ to 2 × 10^17^ cm^−3^. The anodization process is sensitive to the concentration of the free carriers in the used semiconductor substrate. As can be seen from Equation (1), the thickness of the depletion layer decreases as the concentration of electrons increases. As shown in a previous publication [31], the increase of the electron concentration in the ZnSe crystals from 7 × 10^16^ cm^−3^ to (1-2) × 10^18^ cm^−^^3^ allowed us to reduce the pore diameter from 400–500 nm to 40 nm.

### 3.3. Ni Deposition inside the Porous InP Templates

Ni NPs and nanowires (NWs) deposition in the InP template was previously reported [32,33] using an Al_2_O_3_ thin layer to cover the walls of the template pores. In this work, Ni NPs were successfully electrochemically deposited inside the porous InP templates without any preliminary functionalization of the pore walls. To the best of our knowledge, the formation of a direct interface between the walls of porous InP and Ni NPs is here reported for the first time. The InP template with pore diameters of 500 nm was selected since the penetration of solution deep inside the pores is enhanced. At the same time, the larger wall thickness of the InP template provides better mechanical stability in the final structure.

The electrochemical deposition of Ni NPs was performed by the application of pulses with an amplitude of −20 V. It was found that the optimum pulse width (T_on_) was around 8 ms, while the interval between pulses (T_off_) was in the range of seconds, in this way providing enough time for refilling the template pores with the electrolyte solution, and also avoiding massive Ni deposition on the sample surface that could block further metal deposition into pores.

Adjusting the pulse width is essential for a uniform deposition of metals in semiconductor porous templates. An important task consists of avoiding the electrolyte depletion inside pores and refreshing the metal species during the interval between pulses. In our recent paper [34] we found that for uniform deposition along the pore depth (at a pore diameter less than 100 nm) the pulse width should be set at a value of (10–300) µs for enabling the deposition of only 70–80% of the metal ions inside the pores during the applied pulse. This was enough to avoid electrolyte depletion inside pores and the refreshing of the metal species. In the case of InP templates with a pore diameter of 500 nm, the pulse width should be increased to several milliseconds for these purposes. 

**Figure 8:**
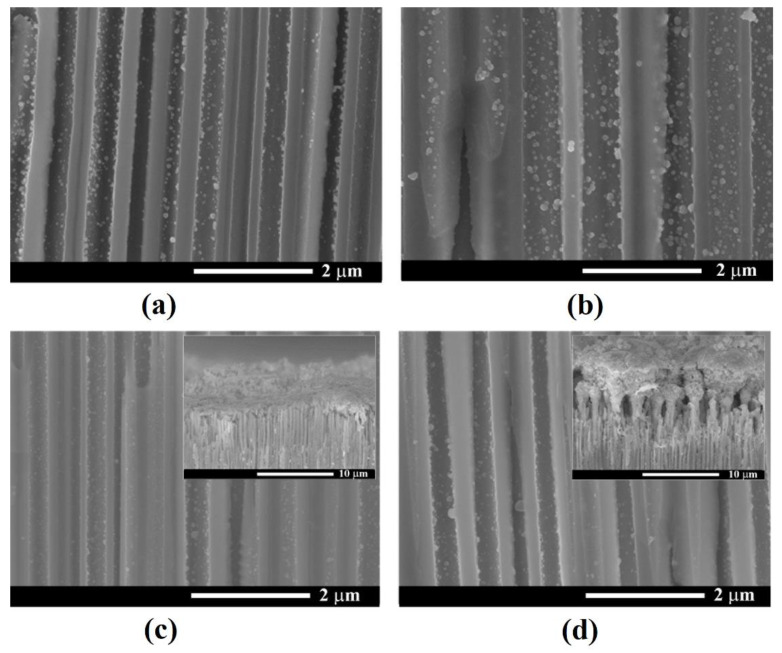
SEM cross-sectional view of Ni NPs deposited in InP template at a constant T_off_ = 1 s and different values of T_on_: (**a**) 4 ms; (**b**) 8 ms; (**c**) 10 ms; (**d**) 15 ms. The inset images in (**c**,**d**) show the upper part of the porous layer, suggesting Ni deposition on the sample surface which blocked the access in the depth of pores.

Figure 8 shows the cross-section images of samples with Ni deposition at a constant T_off_ = 1 s and T_on_ values of 4, 8, 10 and 15 ms. As presented in Figure 8a, applying a pulse width of 4 ms led to the formation of Ni NPs inside the pores. As the T_on_ increased to 8 ms, the diameter of nanoparticles became larger and a slight increase in the density of NPs deposited on the pore walls was also observed. Higher values of T_on_ of 10 and 15 ms, respectively (see the insets from Figure 8c,d), determined the electrodeposition of Ni on the surface of the porous layer, thus blocking any further deposition in the depth of the pores. Therefore, an optimum pulse width of 8 ms to provide Ni NPs growth inside the pores was considered.

**Figure 9:**
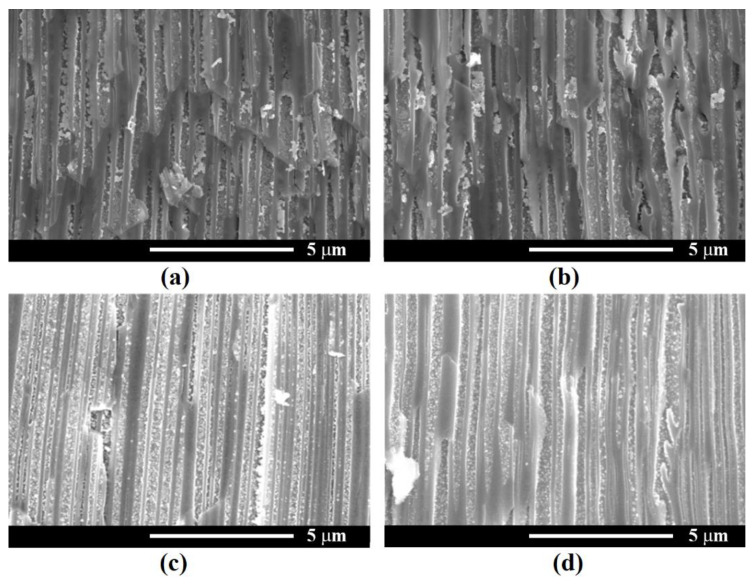
SEM cross-sectional view close to the sample surface of Ni NPs deposited at different T_off_: (**a**) 0.5 s; (**b**) 1 s; (**c**) 1.5 s; (**d**) 2 s.

Figure 9 presents SEM images in cross-sections of samples taken close to the sample surface after deposition at −20 V for different values of T_off_. The deposition was performed with an application of 2500 pulses with T_on_ = 8 ms. The recorded SEM micrographs suggest an optimum T_off_ for a Ni deposition of 1.5 s, which allows a complete covering of the internal surface of nanopores to form nanotubes.

It was observed that the Ni electroplating in the *n*-InP template occurs in agreement with the early reported “hopping electrodeposition” of Au [35]. As can be seen from Figure 8, the deposition of individual Ni nanodots is produced during the electroplating process. In accordance with the proposed model in [35], the nanodots grow up to a critical threshold diameter governed by the Schottky contact height. The deposition is continued by the generation of new nanodots until all internal surfaces are covered by a monolayer of dots. 

The type of the contact, i.e., ohmic or Schottky, can be estimated from the analysis of the difference between the work function of the metal (φ_m_) and the value of the electron affinity of InP (χ_s_). One can expect the formation of a Schottky contact on an *n*-type material when φ_m_ − χ_s_ > 0. Among metals (such as Mg, Zn, Al, Cr, Ni, Pt), Pt exhibits the highest value of φ_m_ − χ_s_ which equals 1.3 eV, followed by Ni (0.81 eV) as seen in Table 1.

The experimental demonstration of the Schottky contact formation is evidenced in Figure 9 by the bright color of the deposited Ni nanodots as compared to the less conductive porous skeleton. As the electroplating process continues, the dots tend to overlap, forming metal nanotube walls.

**Figure 10:**
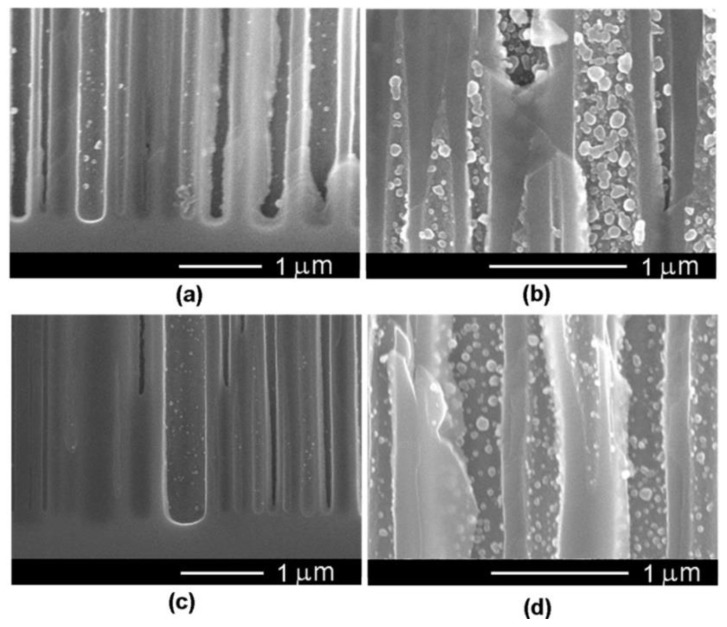
SEM cross-sectional views taken from samples after Ni NPs deposition at the bottom of pores (left column, (**a**,**c**)) and close to the sample surface (right column, (**b**,**d**)) after deposition with T_off_ = 1.5 s (first line, (**a**,**b**)) and T_off_ = 2 s (second line, (**c**,**d**)).

The electrodeposition in this mode results in a gradient-like deposition with maximum metal deposition close to the sample surface and decreasing deposition in the depth of pores, as illustrated in Figure 10.

**Figure 11:**
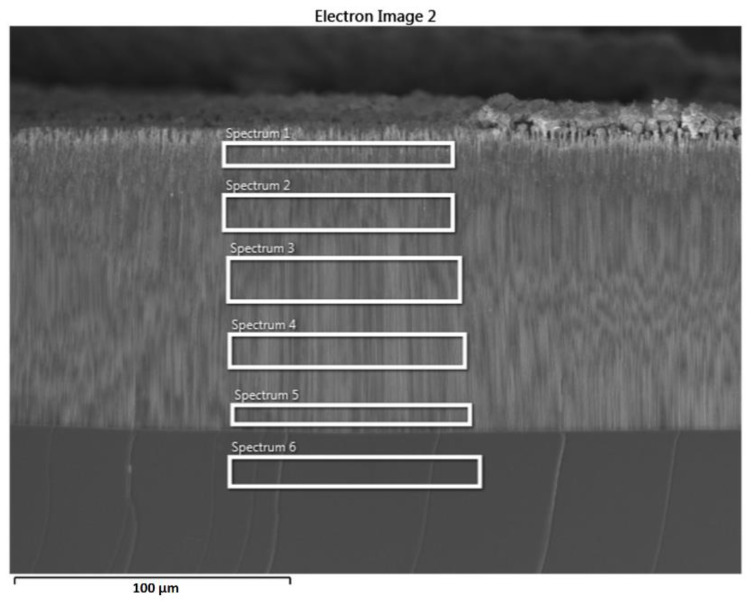
Cross-sectional SEM micrograph denoting the zones for EDX analysis, proving the gradient-like deposition of Ni NPs.

The gradient-like deposition is confirmed by the EDX analysis performed in depth of the porous layer, as shown in Figure 11 and Table 2. As one can see from this analysis, the atomic percentage of Ni decreases from around 17 at.% at the entrance of pores to 2 at.% at the bottom of pores. Such a preferential deposition of Ni NPs in pores close to the surface of the InP template is beneficial for potential applications when interaction with the surrounding environment is desirable.

**Figure 12:**
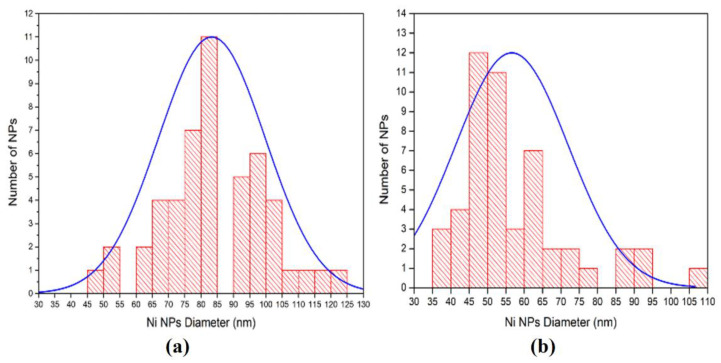
The histograms and corresponding fit of deposited Ni NPs diameters: (**a**) T_off_ =1.5 s; (**b**) T_off_ = 2 s.

The histograms of Ni NPs diameter distribution, as determined from SEM analysis for deposition processes with T_off_ = 1.5 s and T_off_ = 2 s, are shown in Figure 12. This analysis demonstrates that the average Ni NPs diameter is 85 nm in the case of T_off_ = 1.5 s and 55 nm for T_off_ = 2 s. The longer interval between pulses provides better replenishment of the electrolytes inside of pores, thus assuring the nucleation of new nanodots instead of their growing in diameter. It should be noted that the deposition of the size-saturated 20 nm monolayer of gold nanodots electroplated on the InP and GaP porous semiconductor substrates was reported using 10 µs pulse width [35]. These results are in the same range as the data reported in [32], and with diameters of ferromagnetic NPs forming nanotubular structures on GaAs nanowires [23,24,25].

Ni-based nanotubes are promising for magnetic applications, including applications in high-density data storage [20,21]. The technology of ferromagnetic nanotube fabrication is complementary to the previously proposed technology based on GaAs nanowires [24,25]. At the same time, it has the advantages of widening the range of nanotube diameters and diminishing the wall thickness to lower values. Note that nanopores with diameters smaller than 100 nm can be produced in InP templates, while the wall thickness can be reduced thanks to the possibility of depositing a monolayer of metallic nanodots in a controlled fashion. The fabrication of ferromagnetic nanotubes with smaller geometrical parameters is important from the point of view of producing structures with larger magnetic anisotropy. It was previously shown that nanotubes with diameters smaller than 100 nm and thin walls are needed to reach a high magnetic anisotropy [28].

## 4. Conclusions

As a result of the performed investigations, the preparation of uniform porous InP templates presenting the depth of pores up to 100 µm and pore diameter in the range 100–500 nm, depending on the electrical conductivity of the InP substrate, involving an anodization process in an environmentally friendly electrolyte, has been proposed. The optimum composition of the electrolyte was found to be 3.5 M NaCl in water, while the optimum anodization voltage was 6.5 V for substrates possessing a carrier concentration of 2 × 10^18^ cm^−3^ and 20 V for those with a carrier concentration of 2 × 10^17^ cm^−3^. Ni NPs were successfully deposited into prefabricated InP templates without any preliminary surface functionalization or deposition of intermediary layers before pulsed electrodeposition of Ni NPs. The optimum pulse width was found to be around 8 ms, while the interval between pulses was 1.5–2 s. Ni NPs with diameters of 85 nm–55 nm were formed. A gradient-like Ni deposition was demonstrated with predominant deposition in the upper region of the porous template, which is expected to be of interest for interactions with the surrounding environment, i.e., in chemical and biological sensors. The proposed technology could be also prospective for the exploration of magnetic properties and for photocatalytic/photoelectrocatalytic applications.

## Figures and Tables

**Table 1:** The values of the work function for several metals [36] and calculated difference between the work function of the metal and the value of the electron affinity of InP.

Metal	Work Function φ_m_ (eV)	φ_m_ − χ_s_ (eV)
Mg	3.66	−0.72
Zn	3.86	−0.52
Al	4.41	0.03
Cr	4.50	0.12
Au	5.01	0.63
Ni	5.19	0.81
Pt	5.68	1.3

**Table 2:** Chemical composition in atomic percentages versus different positions along the pores according to Figure 11.

Element	Spectrum 1	Spectrum 2	Spectrum 3	Spectrum 4	Spectrum 5	Spectrum 6
P	41.05	42.69	46.38	46.38	46.93	48.30
In	41.52	45.84	49.64	49.82	50.83	51.62
Ni	17.43	11.47	3.98	3.80	2.24	0.08
Total	100.00	100.00	100.00	100.00	100.00	100.00

## Data Availability

The data presented in this study are available on request from the corresponding authors.
